# Mapping light-driven conformational changes within the photosensory module of plant phytochrome B

**DOI:** 10.1038/srep34366

**Published:** 2016-10-03

**Authors:** Silke von Horsten, Simon Straß, Nils Hellwig, Verena Gruth, Ramona Klasen, Andreas Mielcarek, Uwe Linne, Nina Morgner, Lars-Oliver Essen

**Affiliations:** 1 Departments of Chemistry and Biology, Philipps-Universität, D-35032 Marburg, Germany; 2 Institute of Physical and Theoretical Chemistry, Goethe-Universität, D-60438 Frankfurt, Germany; 3 LOEWE Center for Synthetic Microbiology, Philipps-Universität, D-35032 Marburg, Germany

## Abstract

Organisms developed different photoreceptors to be able to adapt to changing environmental light conditions. Phytochromes are red/far-red (r/fr) photochromic photoreceptors that belong to the classical photoreceptors along with cryptochromes and phototropins. They convert absorbed light into a biological signal by switching between two states in a light-dependent manner therefore enabling the light control downstream signalling. Their P_fr_ conformation is the biological active form in plants, but until now only a structure of the ground state (P_r_) was solved. Here, the authors provide information about structural changes occurring during photoconversion within phytochrome B and identify possible interaction sites for its N-terminal extension (NTE) utilising hydrogen/deuterium exchange rate analyses of its amide backbone. Especially, the newly identified light-dependency of two regions in the NTE are of particular interest for understanding the involvement of the phytochrome’s NTE in the regulation of its downstream signalling.

Phytochromes are subdivided in an N-terminal photosensory module (PSM), which is responsible for binding the bilin chromophore and a C-terminal dimerisation module^1^. The PSM of canonical phytochromes consists of a PAS-GAF-PHY domain tripartite module^2^ preceded by an additional N-terminal extension in plant phytochromes. The composition of the dimerisation module is unique for plant phytochromes, consisting of two PAS domains and a kinase-related domain, which function is still unclear. Nevertheless, the module might be involved in downstream signalling by binding of interaction partners^1^,^3^.

Genome sequencing showed that monocot plants harbour three phytochromes (PhyA-PhyC)^4^,^5^ whereas dicots have five phytochromes (PhyA-PhyE)^6^,^7^ which have distinct but also overlapping responsibilities. The five *Arabidopsis* phytochromes are often divided into four sub-families (PhyA, PhyB/D, PhyC and PhyE) based on the high sequence similarity of phytochrome B and D genes due to recent duplication^7^,^8^,^9^. Generally, phytochromes are categorised into two groups: the light-labile type I phytochromes (PhyA) displaying rapid lability in the P_fr_ form and type II phytochromes displaying relative stability in the P_fr_ form (PhyB - PhyE)^6^.

So far, a crystal structure is only available for the PSM of phytochrome B from *Arabidopsis thaliana*, but not for its NTE^10^. This structure reveals high structural similarity to known bacterial phytochromes including the unusual figure-of-eight knot at the PAS-GAF interface, which may serve as binding site for interaction partners^11^, and a long helical spine that links the GAF with the PHY domain. Furthermore, the GAF domain forms the chromophore binding pocket, whereas the PHY domain contributes a long loop protrusion, the tongue region, which closes the chromophore binding pocket by shielding it from bulk solvent access.

The large NTE (M1-T90), unique for PhyB and PhyD, consists of a serine-/glycine rich region^12^ and is crucial for P_fr_ stability^1^,^3^,^13^. Furthermore, the NTE of *At*PhyB contains at least three identified phosphorylation sites (S84, S86, Y104), which are light-dependently dephosphorylated and affect the spectral properties and biological activity. These studies also showed an effect on the binding affinity towards PIF3 (phytochrome interaction factor) upon phosphorylation of these residues^14^,^15^.

Due to their covalently bound chromophore, phytochromes exist in two interconvertible forms, a red light absorbing P_r_ state (biological inactive) and a far-red light absorbing P_fr_ state (biological active)^1^. The latter can light-independently convert back to P_r_ via thermal relaxation. Upon absorption of a photon by the P_r_ ground state, a conversion of the bilin chromophore from the ZZZssa to the ZZEssa configuration of the C15 = C16 double bond takes place^16^,^17^,^18^,^19^, leading to structural changes and thereby inducing the movement to the plant cell nucleus. This transport is considered as the key step in phytochrome signalling^20^. When arrived in the nucleus, phytochromes directly interact with transcription factors such as PIF, promoting their phosphorylation and thereby degradation that causes the activation of light responses^21^,^22^,^23^,^24^,^25^.

Based on a variety of structures from proteobacterial and cyanobacterial relatives^2^,^26^,^27^,^28^,^29^,^30^,^31^,^32^,^33^, especially the structures of both states^34^,^35^, the structural changes during photoconversion could be mapped to the PHY domain, mostly to the tongue region, as well as to the deformation of the helical spine. Within the tongue-GAF interface a tryptophan swap of the conserved ^W^/_F_xE and W^G^/_A_G motifs as well as a local refolding of the tongue upon P_r_→P_fr_ photoconversion was proposed as a general model for phytochrome action^26^,^10^. For a more detailed description see recent reviews on this topic^36^,^37^.

## Results and Discussion

Spectroscopical studies of the PSM of *At*PhyB with PCB as cofactor have shown that the absence of the NTE induces a 10 nm hypsochromic shift in the P_fr_ absorption (see Supplemental Fig. S1a). A similar shift by 7 nm has been reported before for *At*PhyB with PΦB as cofactor^10^. Furthermore, the absence of the NTE enhances the thermal reversion from t/2 = 82.0 min for the WT to 4.3 min for the deletion mutant^10^. We have investigated the influence of the NTE by comparing HDX-MS measurements of the WT with a variant without NTE (ΔNTE). Hydrogen/deuterium exchange mass spectrometry (HDX-MS) offers insights into protein dynamics in solution, an advantage compared to static methods like X-ray crystallography. Due to different accessibilities of the protein backbone amide hydrogens for an exchange with deuterium, information of the environment of these hydrogens can be gained. Also, the system is highly sensitive to structural changes that alter the environment of the hydrogens and therefore their exchange rates^38^,^39^,^40^. A structure is desirable to map the structural context of deuterium uptake, but even without, HDX-MS can provide valuable information, e.g. whether a region is folded or not^41^. We studied *At*PhyB in both states and analysed changes in deuterium exchange in comparison to its P_r_ structure^10^ and a model for its P_fr_ state (see Supplemental Fig. S3). The dark reversion was measured at 5 °C to ensure that at least 75% of the initially formed P_fr_ state are present for the duration of the HDX-measurement (see Supplemental Fig. S1b).

### Dynamic information about *At*PhyB

**Figure 1:**
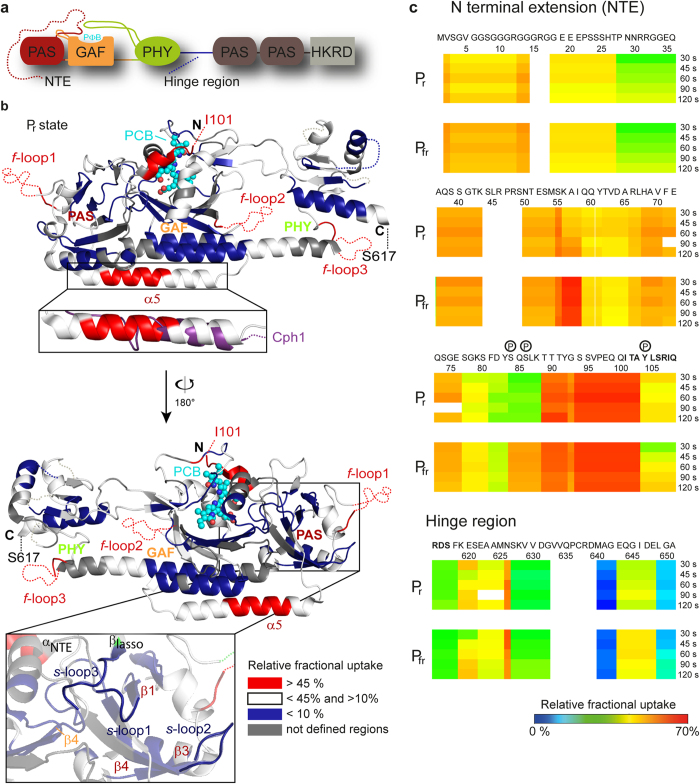
HDX analysis of the (1–651) PSM. (**a**) Domain organisation of PhyB. The binding pocket for the chromophore (PФB = phytochromobilin) is built by the GAF domain and covered by the tongue of the PHY domain and the N terminal extension (NTE). (**b**) The relative fractional uptake after 30 s of the P_r_ state is mapped to the crystal structure (PDB code 4OUR). Strikingly, there are three fast exchanging loops (*f*-loops) and three slow exchanging loops (*s*-loops) on the backside of the PAS domain (lower inlet). The inlay shows the α5_PAS_ helix compared to *Syn*Cph1 (PDB code: 2VEA) is longer but reveals a high exchange rate. (**c**) Deuterium uptake of the NTE and hinge region (615–651) show regions with reduced uptake (P28-Q36, S627-G632, M640-G642 and L649-A651) and light-dependent changes (K56-I58 and S84-K88). Known phosphorylation sites (P) are circled; bold letters indicate the start and end of the *At*PhyB crystal structure; PCB = phycocyanobilin. The nomenclature of secondary structure refers to Burgie *et al*.^10^.

To locate possible contacts between the PSM and the NTE, HDX measurements of the WT and the ΔNTE variant were performed. In order to estimate the quality of our HDX-MS data, we compared the exchange rates with the structure of *At*PhyB. Our data are in good agreement with the structure, as the NTE region shows fast exchange that decreases with the beginning of the PAS domain. Furthermore, three fast exchanging loops, *f*-loop1 (G142-E155), *f*-loop2 (N378-M394) and *f*-loop3 (V464-M471), which are not defined in the *At*PhyB structure proved to be highly flexible and therefore indicate the high quality of the HDX-data (see Fig. 1b).

Differences between our experiments and the structure were found for the α5-helix in the PAS domain. Our experiments show that the N-terminal part of the helix (I228-A238) exerts a very high uptake rate compared to the rest of the protein in the P_r_ state. Interestingly, comparing the crystal structures of *Synechocystis* 6803 Cph1^2^ and *At*PhyB^10^, only the latter harbours an elongated α5-helix (see Fig. 1b, upper inlet), which may not be representative for the solution state of *At*PhyB.

### Light-dependent changes in the NTE

Till now the NTE and the hinge region (R624-R654, see Fig. 1a)^42^, that follows the PSM, were postulated to function as highly flexible regions^1^,^3^. Here, we present contradicting results since several regions exhibit reduced uptakes (P28-Q36, S627-G632, M640-G642 and L649-A651). Interestingly, two regions in the NTE also show light-dependent changes (K56-I58 and S84-K88) (see Fig. 1c). The latter (S84-K88) includes two prominent phosphorylation sites of *At*PhyB, S84 and S86^14^. Likely, structural changes in this region could lead to steric hindrance of the P_fr_ form and explain the increased dark reversion of the phosphomimic variant^14^. Furthermore, in the HDX coverage map a peptide was present without apparent exchange (L67-F71) that is independent of the photostate and may indicate a highly protected region, which is shielded from solvent exposure by protein-protein-interactions (see Supplemental Fig. S3).

### Packing model of the NTE

The backside of the PAS domain harbours three slow exchanging loops, *s*-loop1 (Q115-C119), *s*-loop2 (H193-F201) and *s*-loop3 (K317-V325) (see Fig. 1b, lower inlet). Dimerisation of the proteins as possible explanation for the slow exchanging loops could be excluded, because both *At*PhyB variants show monomeric behaviour in size exclusion chromatography (SEC). Small deviations between the apparent and calculated molecular masses (WT: 102 vs. 72.6 kDa; ΔNTE variant: 81 vs. 63.5 kDa) in the SEC runs most likely derive from the elongated shape of the phytochromes’ photosensory modules (Supplemental Fig. S2a). For further proof, we performed high-resolution native mass spectrometry measurements to validate the oligomerisation state of *At*PhyB^43^. The obtained masses (WT: 72.9 kDa; ΔNTE variant: 63.8 kDa) clearly show that the proteins are monomeric and no indication of dimers could be found (see Supplemental Fig. S2b). Monomeric behaviour of *At*PhyB in solution is in contrast to the parallel dimeric arrangement described before for the ΔNTE structure in a monoclinic crystal form^10^. However, this arrangement *in crystallo* depended significantly on helix bundle formation by the elongated α5-helix of the PAS domain. The monomeric state in solution is hence accompanied by the stronger disorder for the N-terminal part of α5 as found above.

**Figure 2:**
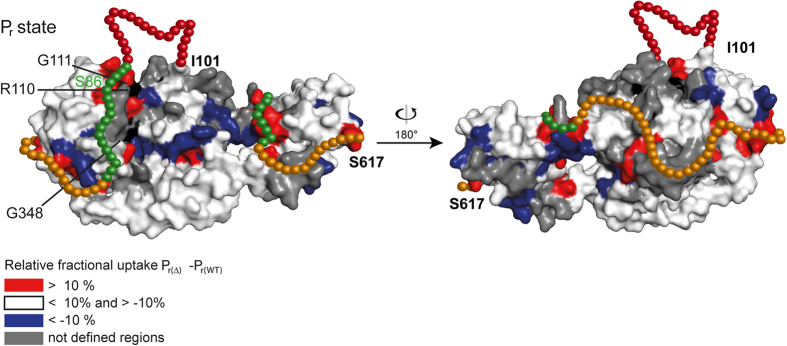
Differences in deuterium uptake of the *At*PhyB wildtype and the ΔNTE variant in the P_r_ state. P_r(Δ)_ − P_r(WT)_ reveals possible interactions sites of the N terminal extension. The NTE is shown as spheres and coloured according to their deuterium exchange rate. Black labelled amino acids are involved in the binding of the phytochrome interaction factor.

Given the high-degree of surface-exposure of the loops we postulate that these regions are instead protected by interactions with the NTE. By analysing the P_r_ state of a ΔNTE variant of *At*PhyB we found indeed differences in deuterium uptake, which allowed us to postulate a packing model between the NTE and the PSM (see Fig. 2).

The fast uptake rates of T89-A103 are indicators of a flexible loop in contrast to the preceding stretch of F81-K88, whose reduced uptake is rationalised due to NTE-PSM-interactions. The S86D mutant, which mimics the phosphorylated state of *At*PhyB at S86, is known to weaken PhyB-PIF interactions. Our data indicate an intimate interaction between this region and the knot region, the putative binding side for PIFs^44^. The lower affinity of phytochromes towards PIF3 in the P_r_ state could be explained by the found NTE-PSM interaction, which blocks part of the binding site.

**Figure 3:**
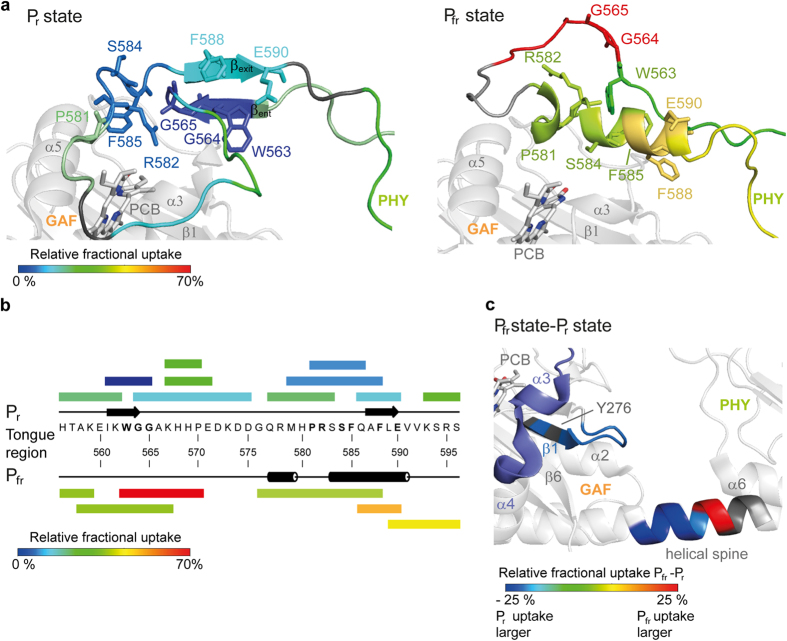
Differences in deuterium uptake of the *At*PhyB wildtype. (**a**) The results for the tongue region of wildtype *At*PhyB are mapped to the P_r_ crystal structure (left) and a P_fr_ model (right), respectively. The largest changes in deuterium uptake are found for the WGG motif, which has a low exchange rate in P_r_ compared to a high one in P_fr_. (**b**) A different digestion pattern refers to structural changes in the tongue region. (**c**) Deuterium uptake difference (P_fr_ − P_r_) near the gap between the GAF-PHY domains. The helical spine as well as a loop and an adjacent beta strand exchanges slower in the P_fr_ state. PCB = phycocyanobilin.

Furthermore, a stretch close to the ^W^/_F_xE (S594-E600) motif of the tongue region apparently interacts with NTE-residues, e.g. P28-Q36, explaining the faster uptake for this motif in the ΔNTE variant. Upon photoconversion the P28-Q36 stretch might switch interaction partners since this region exhibits an increased uptake rate in P_fr_ (see Supplemental Fig. S3). A potential binding partner is the β1_GAF_ strand and its adjacent loop, which become both more protected in the P_fr_ state (see Fig. 3c). Light-dependent differences also occur for the α_NTE_ (Y104-R110), which exerts a reduced uptake rate in the P_fr_ state and may interact with the knot region instead of the chromophore, since the Y104D mutant weakens PIF binding in P_fr_^15^.

### Reorganisation of the GAF and PHY domains in P_fr_

Interestingly, we observed different exchange rates for the two states in the helical spine connecting the GAF and PHY domains. Whereas the L437-M439 stretch exchanges slower, S440-E441 exchange faster in P_fr_ leading to the conclusion that the former region is better protected against the solvent. This indicates state-dependent kinking of the helical spine as already found for bacterial phytochromes^45^,^46^.

The key step during photoconversion is the Z→E isomerization of the double bond during rotation of the D-ring, which presumably initiates conformational changes within the chromophore binding pocket. The regions around residue H276 and P304, which are both involved in forming the aliphatic interface of the D-ring are more protected against deuterium exchange in the P_fr_ state. Likewise, an adjacent loop (F278-E282) exchanges similar slow. Taken together, these findings endorse the assumption of movements or reorganisation of secondary structures in some parts of the GAF and PHY domains resulting in a smaller separation between the two domains, which possibly influences phytochrome downstream signalling (see Fig. 3c).

### Tryptophan swap as general model for phytochromes

Our previously proposed tryptophan swap model that has been evolved from bacterial phytochromes predicts a local refolding of the tongue region, where upon P_r_→P_fr_ conversion the two-stranded β-hairpin is dissolved and an α-helix, interacting with the chromophore binding site, is formed. The largest change in deuterium uptake was indeed found for the W^G^/_A_G motif, which had almost no uptake in P_r_ compared to its high mobility in P_fr_. Its high-degree of protection in the P_r_ state for exchange is in good agreement with structural data, since this motif is located in P_r_ on a β-strand (β_ent_) that packs against the GAF domain, whereas in P_fr_ it is predicted to be part of a highly exposed loop (see Fig. 3a). Furthermore, the proteolytic digestion pattern of the tongue region changes upon the P_r_→P_fr_ transition and supports the transformation of secondary structure and thereby the applicability of the swap model for plant phytochromes (see Fig. 3b).

Overall, our data show that light-triggered structural changes within phytochromes, which cause alterations of their protein-protein interaction pattern, involve a complex orchestration by different regions, including NTE, tongue region and α-helical spine.

## Material and Methods

### Protein Purification

The plasmid carrying the coding sequence for *Arabidopsis thaliana phytochrome B* (1–651; WT) was kindly provided by Andreas Zurbriggen (University Düsseldorf) and the ΔNTE (90–651) lacking the NTE was amplified via the polymerase chain reaction from the plasmid using Phusion^®^ High-Fidelity DNA Polymerase (*NEB*) according to the manufacture’s protocol. Primer (fwd: 5′-CCTCGGACATGTATGACGACGT ACGGTTCC-3′; rv: 5′-GCACGTCTGCAGTTAATGGTGATGGTGATGATG-3′) for the *phyb* sequence were designed to introduce restriction enzyme sites for *Pci*I and *Pst*I and the fragment was cloned into the *Pst*I and *Nco*I restriction sites of the pCDF Duet-1 vector (*Novagen*). The sequences of the plasmids were controlled by dideoxy-sequencing (*GATC*). Cotransformation of the generated plasmid encoding the WT or ΔNTE variant of *At*PhyB with p171^47^ that promotes the *in vivo* biosynthesis of PCB in *E. coli* BL21 Pro (*Clontech*; WT) or *E. coli* BL21 Gold (*Novagen*; ΔNTE) allowed production of holo-PhyB. The expression was carried out in LB medium containing 35 mg/L kanamycin and 100 mg/L ampicillin at 37 °C to an OD_595_ of 0.6. The temperature was then decreased to 18 °C and was induced with 1 mM IPTG and 0.4% arabinose. After 22 h the cells were harvested the culture by centrifugation (8200 g, 15 min, 4 °C) and resuspended in TS buffer (50 mM Tris pH 7.8, 300 mM NaCl, 1 mM β-mercaptoethanol). Bacterial cells were lysed with a French Press (*AMINCO*) and the supernatant was separated by centrifugation (40000 g, 30 min, 4 °C) and followed by Ni^2+^-affinity chromatography (HisTrap^TM^ HP column, *GE*) eluting with TIS buffer (50 mM Tris, 250 mM Imidazol pH 7.8, 300 mM NaCl, 1 mM β-mercaptoethanol). A final purification step was done by size exclusion chromatography (Superdex 200 26/60, *GE*, 2 mL/min) using 50 mM Tris pH 7.8, 5 mM EDTA, 100 mM NaCl, 1 mM β-mercaptoethanol as buffer.

### UV/Vis spectroscopy

UV/Vis spectra were recorded at room temperature with a spectrophotometer V-660 (*Jasco*) using an 1 cm path length cell, a scan speed of 1000 nm/min, a data interval of 0.5 nm and a bandwidth of 1.0 nm. The sample was irradiated for 2 min with red or far-red LEDs (B5-436-30D, *λ*_max_ 664 nm and SMC735, *λ*_max_ 735 nm; both 40 nm FWHM, *Roithner*).

Dark reversion measurements were performed by measuring time-dependent absorbance with a spectropolarimeter J-810 (*Jasco*) and a 2 mm path length cell at 5 °C. After irradiation of the sample for 4 min with red light, the absorbance at 712 nm was recorded every 20 min. Each value corresponds to three separate measurements.

### Native mass spectrometry

Native electrospray ionisation mass spectrometric analysis was performed with a *Waters* Synapt G2-S time-of-flight mass spectrometer equipped with a NanoLockSpray ionisation source. The sample was sprayed using Pd/Pt-coated borosilicate needles prepared in-house. Capillary and cone voltages were set to 2 kV and 150 V, respectively. The trap and transfer collision energies were set to 60 V and 80 V, respectively, with a trap gas flow of 7.0 mL/min.

Directly prior to MS analysis, 30 μL of the protein solution was buffer exchanged into 50 mM Tris pH 7.8, 5 mM EDTA and 1 mM ß-Mercaptoethanol at 4 °C using micro Bio-Spin^®^ columns (*Bio-Rad Laboratories*). 4 μL of the sample solution were loaded into the nanoESI-needle.

### Hydrogen-Deuterium-Exchange-Mass Spectrometry (HDX-MS)

The HDX-mass spectrometric analysis of the samples was carried out using a commercial HDX-automation setup (SYNAPT G2-Si, *Waters*) including a two-arm robotic autosampler (*LEAP Technologies*), an ACQUITY UPLC M-Class system (*Waters*) and HDX manager (*Waters*). The samples were transferred by PD-10 into a low salt buffer (10 mM Tris pH 7.8, 100 mM NaCl, 1 mM β-mercaptoethanol), centrifuged for 10 min at 16100 g and 4 °C prior to irradiation with 656 nm (P_fr_ state) or 735 nm (P_r_ state) for 4 min in darkness, afterwards wrapped in aluminium foil and cooled to 1 °C. For each LCMS run, 7.5 μL of the protein solution (60 μM) were pipetted in a fresh vial of the exchange plate at 25 °C and diluted with 61.8 μL of either H_2_O-buffer (t0-runs) or D_2_O-buffer (exchange runs). After incubation for pre-defined times, 55 μL of this solution were transferred to a fresh quench vial containing 55 μL of quenching solution (400 mM H_3_PO_4_/KH_2_PO_4_ pH 2.2), which was pre-dispensed and pre-cooled to 1 °C for 10 minutes before starting the first run. After quenching, 95 μL of the resulting solution was immediately injected into the pepsin column (HDX manager, *Waters*).

Digestion was done online using an Enzymate BEH pepsin column (*Waters*) at 20 °C with water/0.1% formic acid at a flow rate of 100 μL/min. Subsequently, peptic peptides were trapped at 0.5 °C using a C18 trap column. Separation of peptides was achieved at 0.5 °C utilising a 1 × 100 mm ACQUITY UPLC BEH C18 1.7 μm column (*Waters*) at a flow rate of 30 μL/min with the following gradient of solvents A (water 0.1% formic acid) and B (acetonitrile, 0.1% formic acid): Linear increase from 5% B to 35% B within 7 minutes, followed by a ramp to 85% B within 1 minute and holding 85% B for additional 2 minutes. Finally, the column was washed at 95% for 1 minute and re-equilibrated to 5% B for 5 minutes. During separation of peptides using the chromatographic column, the pepsin column was washed by injecting 3 times 80 μL of 4% acetonitrile and 0.5 M guanidinium chloride.

Enhanced high definition MS (HDMSe) mode was used for t0 peptide detection, which is a workflow provided by Waters for data independent acquisition, including ion mobility separation (IMS) of precursor ions within the gas phase and alternating lower and higher energies applied to the transfer cell (higher energies lead to fragmentation of IMS separated precursor ions, lower energies result in non-fragmented peptide molecular ion spectra), and HDMS (also including IMS, but with only lower energies applied to the transfer cell preventing fragmentation) for measuring exchanged peptides. Lock mass spectra were measured every 45 seconds using Glu-fibrinopeptide B as standard (M^2+^ = 785.8427 m/z). Blank runs were performed between each sample to avoid peptide carryover from previous runs.

t0 peptide identification was performed using ProteinLynx Global SERVER 3.0.1 (*Waters*) with custom-created databases and the setting “no enzyme”. Final assignment of deuterium incorporation was done with DynamX 3.0 (*Waters*). The minimum peak intensity was set to 10^3^ counts and a peptide length between four and 15 was chosen. Moreover, tolerances of 0.5 min for the retention time and 25 ppm for m/z values were applied for the peptide assignment, generating an overall sequence coverage of 91% for the WT and 84% for the ΔNTE. For the WT 168 peptides were analysed in P_r_ and 165 in P_fr_ with an overall redundancy of 2.3 per amino acid, whereas for the ΔNTE variant 153 peptides were analysed with an redundancy of 2.3. A standard deviation of 4 σ was used to quantify the amount of variation between the repetitions. Statistically significant differences in deuterium uptake were determined by performing a two-sided t-test at a 98% confidence interval^48^ (see Supplementary Table S1 and S2). Additionally, the assumption of homogeneity of variances was tested prior by Levene’s F test.

The results of the analysis were mapped on the structure of the P_r_ state of *Arabidopsis thaliana* PhyB (PDB code: 4OUR)^10^ or a model of the P_fr_ state. The latter is generated by MODELLER 9.10^49^ using a hybrid template consisting of the crystal structure of *At*PhyB, the PHY domain of *Deinococcus radiodurans* BphP (PDB code: 4O01)^35^ and the tongue region of *Pseudomonas aeruginosa* BphP (PDB code: 3NHQ)^34^. Figures were created using PYMOL 1.6 (*DeLano Scientific*).

## Additional Information

**How to cite this article**: Horsten, S. v. *et al*. Mapping light-driven conformational changes within the photosensory module of plant phytochrome B. *Sci. Rep*. **6**, 34366; doi: 10.1038/srep34366 (2016).

## Supplementary Material

Supplementary Information
