# Supplementary material for: Mapping light-driven conformational changes within the photosensory module of plant phytochrome B
Source: Sci Rep. 2016 Oct 3;6:34366. doi: 10.1038/srep34366 (PMC5046071; doi:10.1038/srep34366)
Supplement: Supplementary Information [file srep34366-s1.pdf]

Supplemental information for:

Mapping light-driven conformational changes within the photosensory module of plant phytochrome B

Silke von Horsten[1], Simon Straß[1], Nils Hellwig[2], Verena Gruth[1], Ramona Klasen[1], Andreas Mielcarek[1], Uwe Linne[1], Nina Morgner[2], Lars-Oliver Essen[1,3*]

[1]Departments of Chemistry and Biology, Philipps-Universität, D-35032 Marburg, Germany

[2]Institute of Physical and Theoretical Chemistry, Goethe-Universität, D-60438 Frankfurt, Germany.

[3]LOEWE Center for Synthetic Microbiology, Philipps-Universität, D-35032 Marburg, Germany

Figure S1: Spectroscopical characterisation of PCB-bound *At*PhyB WT and its variant without N-terminal extension (ΔNTE). (a) UV/Vis absorption spectra of the WT and the ΔNTE variant after far-red (black curve) and red light (red curve) irradiation; the difference spectrum, A[P_r]-A[P_{fr}], is shown in blue. (b) Dark reversion kinetics were measured in darkness at 5 °C for the Pfr→Pr conversion of the wildtype.

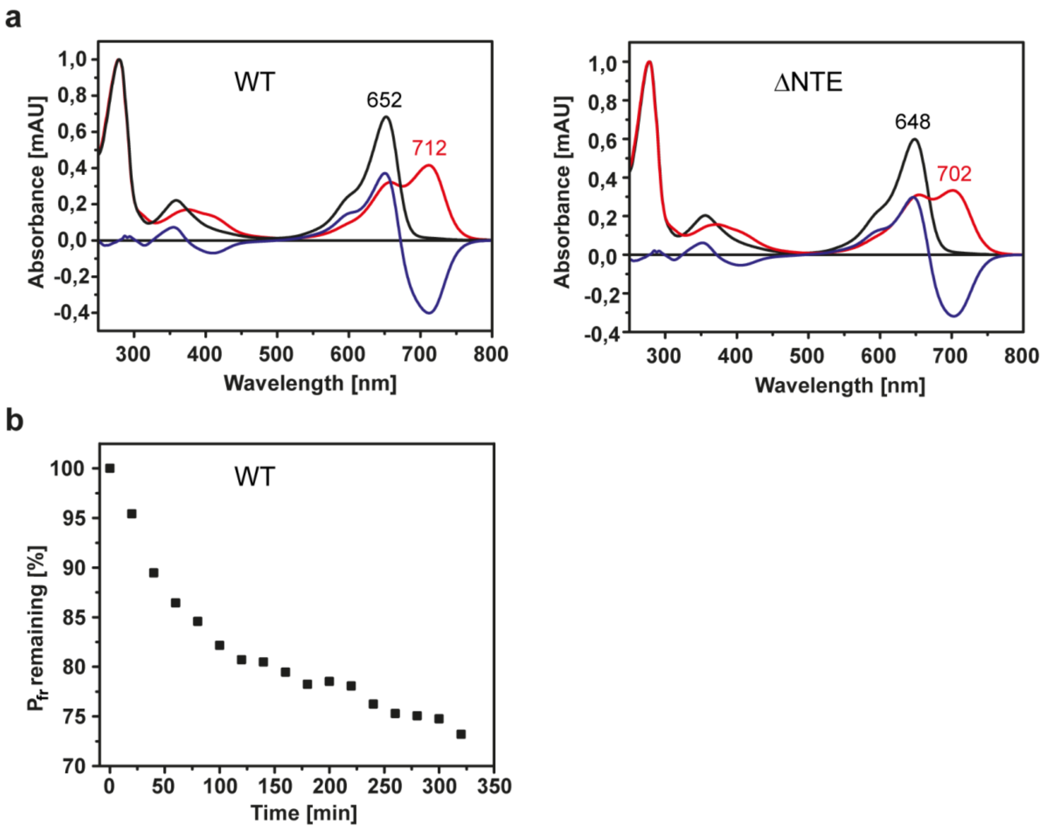

Figure S2: Characterisation of the oligomerisation state of PCB-bound *At*PhyB WT and ΔNTE. (a) Elution profile of the size exclusion chromatography for the WT and the ΔNTE variant measured at 280 nm (blue), 650 nm (black) and 700 nm (red). Small deviations between the apparent and calculated molecular masses (WT: 102 vs. 72.6 kDa; ΔNTE variant: 81 vs. 63.5 kDa) in the SEC runs most likely derive from the elongated shape of the phytochromes' photosensory modules. The green line indicates the calibration of the column with standard proteins (b) Native mass spectrometry measurements. Deconvolution of the m/z distribution shows the existence of a monomeric state for both *At*PhyB variants. The absence of signals between the monomeric signals clearly indicate that no dimers are present. A second signal set at a m/z of 3000 can be assigned to denatured protein, since it also has a mass of 72.9 kDa.

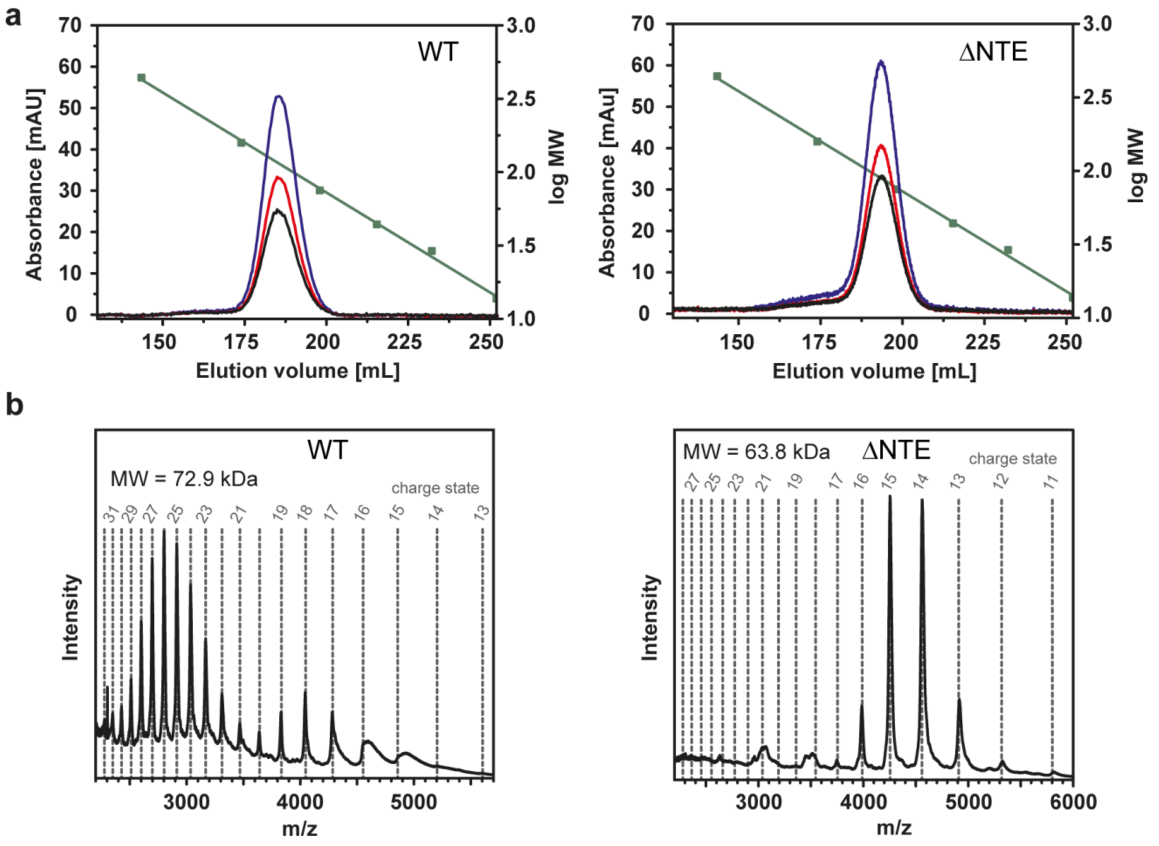

Figure S3: Coverage map for the P_r and the difference between P_{fr} and P_r of the WT after 30 s deuterium incubation. Each box reflects one peptide and the coloring is according to the relative fractional deuterium uptake. Due to a different proteolytic digestion pattern in the tongue region (S555-R595, black box), the difference in deuterium uptake can not be mapped per peptide.

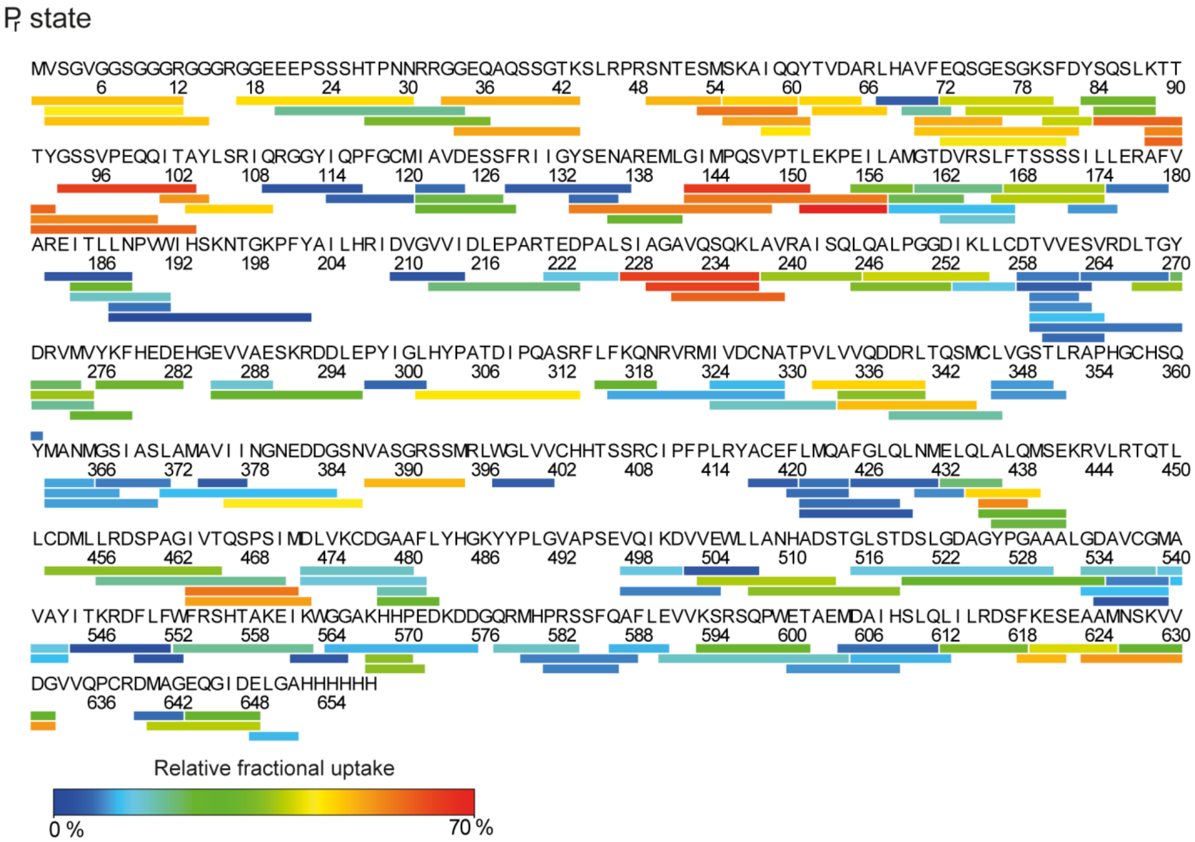
Pr state
MVSGVGGSGGGRGGGRGGEEEPSSSHTPNNRRGGEQAQSSGTKSLRPRSNTESMSKAIQQYTVDARLHAVFEQSGESGKSFDYSQSLKTT
6 12 18 24 30 36 42 48 54 60 66 72 78 84 90
TYGSSVPEQQITAYLSRIQRGGYIQPFGCMIAVDESSFRIIGYSENAREMLGIMPQSVPTLEKPEILAMGTDVRSLFTSSSSILLERAFV
96 102 108 114 120 126 132 138 144 150 156 162 168 174 180
AREITLLNPVWIHSKNTGKPFYAILHRIDVGVVIDLEPARTEDPALSIAGAVQSQKLAVRAISQLQALPGGDIKLLCDTVVESVRDLTGY
186 192 198 204 210 216 222 228 234 240 246 252 258 264 270
DRVMVYKFHEDEHGEVVAESKRDDLEPYIGLHYPATDIPQASRFLFKQNRVRMIVDCNATPVLVVQDDRLTQSMCLVGSTLRAPHGCHSQ
276 282 288 294 300 306 312 318 324 330 336 342 348 354 360
YMANMGSIASLAMAVIINGNEDDGSNVASGRSSMRLWGLVVCHHTSSRCIPFPLRYACEFLMQAFGLQLNMELQLALQMSEKRVLRTQTL
366 372 378 384 390 396 402 408 414 420 426 432 438 444 450
LCDMLLRDSPAGIVTQSPSIMDLVKCDGAAFLYHGKYYPLGVAPSEVQIKDVVEWLLANHADSTGLSTDSLGDAGYPGAAALGDAVCGMA
456 462 468 474 480 486 492 498 504 510 516 522 528 534 540
VAYITKRDFLFWFRSHTAKEIKWGGAKHHPEDKDDGQRMHPRSSFQAFLEVVKSRSQPWETAEMDAIHSLQLILRDSFKESEAAMNSKVV
546 552 558 564 570 576 582 588 594 600 606 612 618 624 630
DGVVQPCRDMAGEQGIDELGAHHHHHH
636 642 648 654
Relative fractional uptake
0 %
70 %

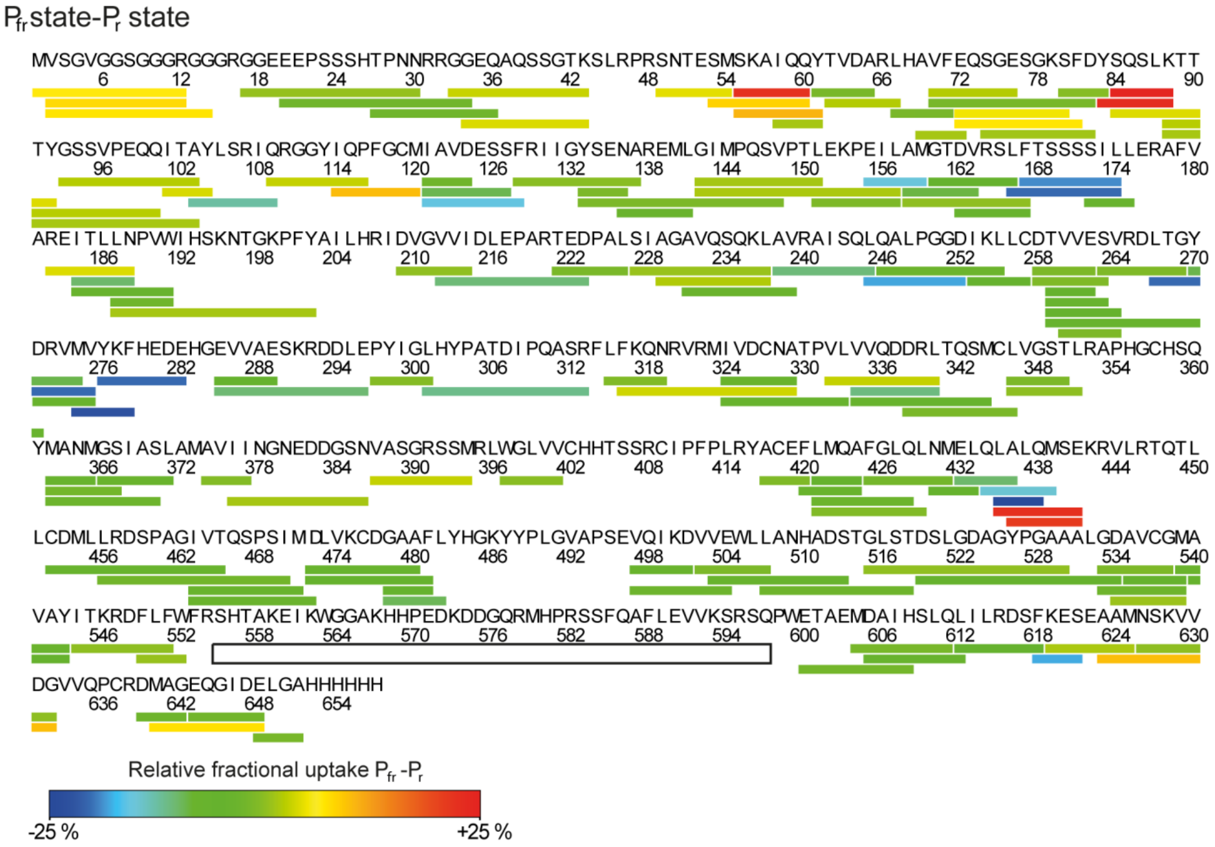
Pfr state-Pr state
MVSGVGGSGGGRGGGRGGEEEPSSSHTPNNRRGGEQAQSSGTKSLRPRSNTESMSKAIQQYTVDARLHAVFEQSGESGKSFDYSQSLKTT
6 12 18 24 30 36 42 48 54 60 66 72 78 84 90
TYGSSVPEQQITAYLSRIQRGGYIQPFGCMIAVDESSFRIIGYSENAREMLGIMPQSVPTLEKPEILAMGTDVRSLFTSSSSILLERAFV
96 102 108 114 120 126 132 138 144 150 156 162 168 174 180
AREITLLNPVWIHSKNTGKPFYAILHRIDVGVVIDLEPARTEDPALSIAGAVQSQKLAVRAISQLQALPGGDIKLLCDTVVESVRDLTGY
186 192 198 204 210 216 222 228 234 240 246 252 258 264 270
DRVMVYKFHEDEHGEVVAESKRDDLEPYIGLHYPATDIPQASRFLFKQNRVRMIVDCNATPVLVVQDDRLTQSMCLVGSTLRAPHGCHSQ
276 282 288 294 300 306 312 318 324 330 336 342 348 354 360
YMANMGSIASLAMAVIINGNEDDGSNVASGRSSMRLWGLVVCHHTSSRCIPFPLRYACEFLMQAFGLQLNMELQLALQMSEKRVLRTQTL
366 372 378 384 390 396 402 408 414 420 426 432 438 444 450
LCDMLLRDSPAGIVTQSPSIMDLVKCDGAAFLYHGKYYPLGVAPSEVQIKDVVEWLLANHADSTGLSTDSLGDAGYPGAAALGDAVCGMA
456 462 468 474 480 486 492 498 504 510 516 522 528 534 540
VAYITKRDFLFWFRSHTAKEIKWGGAKHHPEDKDDGQRMHPRSSFQAFLEVVKSRSQPWETAEMDAIHSLQLILRDSFKESEAAMNSKVV
546 552 558 564 570 576 582 588 594 600 606 612 618 624 630
DGVVQPCRDMAGEQGIDELGAHHHHHH
636 642 648 654
Relative fractional uptake Pfr -Pr
-25 %
+25 %

Table S1: Selected HDX peptides with raw values and standard deviations (SD). Mean relative deuterium uptake for P_r and P_{fr} of the WT and P_r of the Δ variant for an incubation time of 30 s as well as the percentage uptake.

Peptide Start	End	MaxD	Protein	State	Uptake /Da	Uptake SD /Da	Uptake /%
27	36	8	WT	P_{fr}	2.3	0.0	29
27	36	8	WT	P_r	2.3	0.0	29
55	60	5	WT	P_{fr}	3.5	0.0	70
55	60	5	WT	P_r	2.3	0.1	46
81	87	6	WT	P_{fr}	4.4	0.1	73
81	87	6	WT	P_r	3.5	0.0	58
83	88	5	WT	P_{fr}	2.7	0.1	54
83	88	5	WT	P_r	1.5	0.1	25
103	109	6	WT	P_{fr}	2.0	0.2	33
103	109	6	WT	P_r	2.8	0.1	47
114	120	5	WT	P_{fr}	0.6	0.0	12
114	120	5	WT	P_r	0.2	0.1	4
108	120	5	Δ	P_r	1.7	0.0	15
121	124	3	WT	P_{fr}	0.0	0.0	0
121	124	3	WT	P_r	0.1	0.1	4
121	128	7	Δ	P_r	1.3	0.0	19
128	137	9	WT	P_{fr}	0.2	0.1	2
128	137	9	WT	P_r	0.2	0.0	2
133	136	3	WT	P_{fr}	0.1	0.1	2
133	136	3	WT	P_r	0.1	0.1	4
142	157	12	WT	P_{fr}	7.0	0.1	58
142	157	12	WT	P_r	6.8	0.2	57
151	157	5	WT	P_{fr}	3.5	0.1	70
151	157	5	WT	P_r	3.5	0.1	70
172	175	3	WT	P_r	0.2	0.0	6
168	174	6	Δ	P_r	1.6	0.0	27
187	202	13	WT	P_{fr}	0.0	0.1	0
187	202	13	WT	P_r	0.0	0.1	0
192	203	10	Δ	P_r	2.3	0.0	23
209	214	5	WT	P_r	0.0	0.0	0
203	211	8	Δ	P_r	1.4	0.1	18
213	223	9	Δ	P_r	1.4	0.0	16
227	237	10	WT	P_{fr}	6.5	0.1	65
227	237	10	WT	P_r	6.4	0.1	64
231	239	8	WT	P_{fr}	4.5	0.1	56
231	239	8	WT	P_r	4.8	0.1	60

Peptide Start	End	MaxD	Protein	State	Uptake /Da	Uptake SD /Da	Uptake /%
267	275	8	WT	P_{fr}	1.2	0.1	15
267	275	8	WT	P_r	2.8	0.1	35
276	282	6	WT	P_{fr}	0.6	0.1	8
276	282	6	WT	P_r	1.8	0.1	30
297	301	3	WT	P_r	0.1	0.0	3
299	313	13	Δ	P_r	3.3	0.1	25
301	313	10	WT	P_{fr}	3.3	0.0	33
301	313	10	WT	P_r	4.4	0.1	44
316	329	13	WT	P_{fr}	1.2	0.3	11
316	329	13	WT	P_r	1.0	0.1	8
338	346	8	WT	P_r	1.2	0.0	15
338	344	6	Δ	P_r	3.6	0.1	61
340	346	5	Δ	P_r	2.6	0.0	51
371	384	13	WT	P_r	1.1	0.1	8
373	386	13	Δ	P_r	4.4	0.1	34
376	386	9	WT	P_{fr}	4.5	0.1	50
376	386	9	WT	P_r	4.3	0.1	48
387	394	7	WT	P_{fr}	3.7	0.1	52
387	394	7	WT	P_r	3.4	0.1	49
434	439	5	WT	P_{fr}	1.6	0.0	32
434	439	5	WT	P_r	2.3	0.1	46
435	441	6	WT	P_{fr}	2.9	0.0	48
435	441	6	WT	P_r	1.6	0.0	27
463	471	7	WT	P_{fr}	3.5	0.1	50
463	471	7	WT	P_r	4.1	0.1	57
558	567	9	WT	P_{fr}	2.1	0.1	23
589	601	11	WT	P_r	3.2	0.2	29
590	604	13	WT	P_r	1.7	0.1	13
589	601	11	Δ	P_r	6.2	0.1	56
589	603	13	Δ	P_r	7.2	0.0	55
563	570	6	WT	P_{fr}	5.4	0.1	90
561	565	4	WT	P_r	0.0	0.0	0
612	618	6	WT	P_r	1.9	0.1	32
613	625	12	Δ	P_r	6.4	0.1	53
